# Oxidative Stress Is Associated with Neuroinflammation in Animal Models of HIV-1 Tat Neurotoxicity

**DOI:** 10.3390/antiox3020414

**Published:** 2014-05-16

**Authors:** Jean-Pierre Louboutin, Lokesh Agrawal, Beverly A. S. Reyes, Elisabeth J. Van Bockstaele, David S. Strayer

**Affiliations:** 1 Department of Pathology, Anatomy and Cell Biology, Thomas Jefferson University, Philadelphia, PA 19107, USA; E-Mails: lokagrawal@gmail.com (L.A.); David.Strayer@jefferson.edu (D.S.S.); 2 Department of Neurosurgery, Farber Institute for Neurosciences, Thomas Jefferson University, Philadelphia, PA 19107, USA; E-Mails: beverly.reyes@drexelmed.edu (B.A.S.R.); elisabeth.vanbockstaele@drexelmed.edu (E.J.B.)

**Keywords:** HIV-1, Tat, oxidative stress, brain, antioxidant enzymes, gene therapy, SV40, neuroinflammation, microglia, astrocytes

## Abstract

HIV-1 *trans*-acting protein Tat, an essential protein for viral replication, is a key mediator of neurotoxicity. If Tat oxidant injury and neurotoxicity have been described, consequent neuroinflammation is less understood. Rat caudate-putamens (CPs) were challenged with Tat, with or without prior rSV40-delivered superoxide dismutase or glutathione peroxidase. Tat injection caused oxidative stress. Administration of Tat in the CP induced an increase in numbers of Iba-1- and CD68-positive cells, as well as an infiltration of astrocytes. We also tested the effect of more protracted Tat exposure on neuroinflammation using an experimental model of chronic Tat exposure. SV(Tat): a recombinant SV40-derived gene transfer vector was inoculated into the rat CP, leading to chronic expression of Tat, oxidative stress, and ongoing apoptosis, mainly located in neurons. Intra-CP SV(Tat) injection induced an increase in microglia and astrocytes, suggesting that protracted Tat production increased neuroinflammation. SV(SOD1) or SV(GPx1) significantly reduced neuroinflammation following Tat administration into the CP. Thus, Tat-induced oxidative stress, CNS injury, neuron loss and inflammation may be mitigated by antioxidant gene delivery.

## 1. Introduction

Under normal physiologic conditions, reactive oxygen species (ROS), which include superoxide (O_2_^−^), hydrogen peroxide (H_2_O_2_) and hydroxyl radical (OH^−^), are generated at low levels and play important roles in signaling and metabolic pathways [1]. ROS levels are controlled by antioxidants such as superoxide dismutases (SOD), glutathione peroxidase (GPx1), glutathione and catalase. The tripeptide glutathione (γ-l-glutamyl-l-cysteinylglycine, GSH) is the key low molecular thiol antioxidant involved in the defense of brain cells against oxidative stress. Oxidative stress arises due to the disturbances of the balance in pro-oxidant/antioxidant homeostasis that further causes the generation of ROS which are potentially toxic for neurons.

Abnormalities in oxidative metabolism have been reported in many nervous system diseases. These include neurodegenerative diseases (Parkinson’s disease, Alzheimer’s disease, Huntington’s disease, amyotrophic lateral sclerosis and cerebellar degeneration) [2,3,4,5,6], vascular diseases (ischemia-reperfusion) [7] or toxic reactions (chronic alcoholism) [8], as well as aging [9].

Similarly, neuroinflammation plays an important role in the pathophysiology of numerous chronic neurodegenerative diseases like Parkinson’s disease (PD), and Alzheimer’s disease (AD) [10,11]. Neuroinflammation is also observed in acute brain insults like stroke and status epilepticus (SE) [12,13,14]. Activation of microglial cells, the brain’s resident phagocytes, which both produce and respond to proinflammatory factors during the inflammation process, might result in subsequent neurodegeneration. Anti-inflammatory treatment can protect from onset or progression of AD in patients [15].

Oxidative stress and neuroinflammation are involved as well in Human Immunodeficiency Virus-1-(HIV-1)-associated neurocognitive disorder (HAND) [16]. HAND covers a range of HIV-related CNS dysfunction. HIV-1 enters the Central Nervous System (CNS) soon after it enters the body. There, it is largely impervious to highly active anti-retroviral therapeutic drugs (HAART). The brain may be an important reservoir for the virus, and neurodegenerative and neuroinflammatory changes may continue despite use of HAART [17,18,19]. As survival with chronic HIV-1 infection improves, the number of people harboring the virus in their CNS increases. The prevalence of HAND therefore continues to rise. HAND remains a significant independent risk factor for AIDS mortality [17,18,19,20].

In the brain, HIV-1 mainly infects microglia and perivascular macrophages, and rare astrocytes, leading to increased production of cytokines, such as interleukin-6 (IL-6), IL-1β and tumor necrosis factor-α (TNF-α), and chemokines such as monocyte chemotactic protein-1 (MCP-1) [21]. Macrophages and microglia release HIV-1 proteins, several of which are neurotoxins, including the envelope (Env) protein gp120 and Tat [22,23,24,25]. Neurons themselves are rarely infected by HIV-1, and neuronal damage is mainly indirect. HAND involves ROS-mediated damage to cellular protein and lipids, and neuronal apoptosis.

The pathogenesis of HAND largely reflects the neurotoxicity of HIV-1 proteins. The HIV-1 *trans*-acting protein Tat, an essential protein for viral replication, is a key mediator of neurotoxicity. Brain areas that are particularly susceptible to Tat toxicity include the CA3 region and the dentate gyrus of the hippocampus and the striatum. Tat is internalized by neurons primarily through lipoprotein related protein receptor (LRP) and by activation of NMDA receptor [26]. It also interacts with several cell membrane receptors, including integrins, VEGF receptor in endothelial cells and possibly (C-X-C motif) receptor 4 (CXCR4) [27].

Tat can promote excitotoxic neuronal apoptosis [28,29] by activating endoplasmic reticulum pathways to release intracellular calcium ([Ca^2+^]i) [30]. Consequent dysregulation of calcium homeostasis [28,31,32] leads to mitochondrial calcium uptake, caspase activation and, finally, neuronal death. Tat can directly depolarize neuron membranes, independently of Na^+^ flux [33] and may potentiate glutamate- and NMDA-triggered calcium fluxes and neurotoxicity [32]. Tat also triggers lipid peroxidation [29] by generating the ROS superoxide (O_2_^−^) and hydrogen peroxide (H_2_O_2_). It activates inducible nitric oxide synthase (iNOS) to produce nitric oxide (NO), which binds superoxide anion to form the highly reactive peroxynitrite (ONOO^−^) [34]. The latter may attack lipids, proteins and DNA, enhancing oxidant-related injury.

Tat neurotoxicity has been reported in cultured cells*,* but few studies have demonstrated its neurotoxic properties *in vivo* [24,35,36,37]. Tat-induced protein oxidation is well documented [36,37] but little is known about its effects on lipid peroxidation. We demonstrated that Tat activates multiple signaling pathways. In one of these, Tat-induced superoxide acts as an intermediate, while the other utilizes peroxide as a signal transducer [38]. We asked here if direct injection of Tat into the caudate-putamen (CP) can induce neuroinflammation and oxidative stress. We also tested the consequences of protracted exposure to Tat, by expressing Tat over time in neurons. Finally, we assessed the effects of prior gene delivery of antioxidant enzymes Cu/Zn superoxide dismutase (SOD1) or glutathione peroxidase (GPx1) into the CP before injecting Tat.

## 2. Material and Methods

### 2.1. Animals

Female Sprague-Dawley rats (300–350 g) were purchased from Charles River Laboratories (Wilmington, MA, USA). Protocols for injecting and euthanizing animals were approved by the Thomas Jefferson University Institutional Animal Care and Use Committee (IACUC), and are consistent with Association for Assessment and Accreditation of Laboratory Animal Care (AAALAC) standards. Because estrogens can influence inflammation in the brain and attenuate Tat-induced oxidative stress, experiments were done in female rats at similar points of their estrous cycle determined by vaginal smears. Animals were preferably injected during the diestrus stage of the estrous cycle. Oestrogens are typically low during this stage. In any case, animals were not injected during the estrus stage of the cycle when oestrogens levels are elevated. The diet that the animals received was a standard commercial, regular powdered rodent diet without any component that might cause oxidative stress (e.g., high fat diet, or high manganese) and was not folate/methyl or iron deficient. Animals had free access to water and diet. Numbers of animals used in experiments are indicated in the “Experimental Design” section.

### 2.2. Reagents

Recombinant Tat was obtained through the NIH AIDS Research and Reference Reagent Program, Division of AIDS, NIAID, NIH, Germantown, MD. Tat was reconstituted in PBS containing 1 mg/mL BSA and 0.1 mM DTT, then aliquoted and stored in dark bottles to avoid oxidation. Endotoxin levels of Tat was also determined using E-Toxate kit (ET0200) (Sigma Chemical Co., St Louis, MO, USA). Tat protein was found to be endotoxin free with concentration below 0.015 Endotoxin units (EU)/mL, which was below the lowest concentration of endotoxin standard.

### 2.3. Antibodies

Diverse primary antibodies were used: rabbit anti-Iba1 (IgG; 1:100), a marker of quiescent and active microglia (Waco Chemicals, Osaka, Japan), mouse anti-glial fibrillary acidic protein (GFAP) (IgG2b; 1:100) (BD Pharmingen Franklin Lakes, NJ, USA), mouse anti-neuN (IgG1; 1:100), mouse anti-rat CD68/ED1 (IgG1; 1:100), a marker of activated microglial cells in a phagocytic state (Serotec, Oxford, UK). Secondary antibodies were used at 1:100 dilution: FITC and Tetramethyl Rhodamine IsoThioCyanate (TRITC)-conjugated goat anti-mouse IgG (γ-chain specific and against whole molecule respectively), TRITC-conjugated goat anti-rabbit IgG (whole molecule), FITC-conjugated sheep anti-rabbit IgG (whole molecule) (Sigma, Saint-Louis, MO, USA), FITC and TRITC-conjugated donkey anti-mouse IgG (whole molecule), FITC and TRITC-conjugated donkey anti-sheep IgG (whole molecule), Cy3-conjugated donkey anti-rabbit IgG (whole molecule) and anti-goat IgG (whole molecule) (Jackson ImmunoResearch Laboratories, Inc., WestGrove, PA, USA).

### 2.4. Vector Production

The general principles for making recombinant, *Tag-*deleted, replication-defective SV40 viral vectors have been previously reported [39,40]. Cu/Zn superoxide dismutase (SOD1) or glutathione peroxidase (GPx1) transgenes were subcloned into pT7[RSVLTR], in which transgene expression is driven by the Rous Sarcoma Virus long terminal repeat (RSV-LTR). Tat expression in SV(Tat) is driven by RSV-LTR. The cloned rSV40 genome was excised from its carrier plasmid, gel-purified and recircularized, then transfected into COS-7 cells. These cells supply large T-antigen (Tag) and SV40 capsid proteins *in trans*, which are needed to produce recombinant replication-defective SV40 viral vectors [41]. Crude virus stocks were prepared as cell lysates, then band-purified by discontinuous sucrose density gradient ultracentrifugation and titered by quantitative (Q)-PCR [42]. SV(human bilirubin-uridine 5′-diphosphate-glucuronosyl-transferase) (BUGT), which was used here as negative control vector, with a non-toxic byproduct, has been reported [43].

### 2.5. Experimental Design

#### 2.5.1. Tat Injection

It is difficult to know what concentration of Tat in the brain is sufficient to produce pathogenesis [24]. The amount of Tat injected was decided after previous experiments showing that 10 ng Tat was the optimal amount of Tat necessary for inducing neuronal apoptosis *in vitro* [38]. In this previous study, cells were incubated with 0, 1, 10 and 100 ng/mL of recombinant soluble Tat for 48 h. Apoptotic bodies were analyzed using terminal deoxynucleotidyl transferase-mediated nick end labeling (TUNEL). Neurons were identified by their expression of MAP-2. The intensity and frequency of TUNEL+ cells were significantly higher (*p* < 0.001) as compared to untreated cultures. Increasing Tat concentration above 10 ng/mL did not increase apoptosis significantly. Thus, 10 ng/mL of Tat was used in all subsequent *in vitro* studies [38].

For *in vivo* studies, Tat was suspended in saline at a concentration of 10 ng/μL. The volume of solution containing Tat that was injected into the striatum was 1 μL. By comparison, the concentration of Tat injected in the striatum ranged from 1–50 μg/μL [24], to 20 μg/μL [44], and 50 μg/μL [37].

In order to study Tat-induced abnormalities, 1 μL saline containing 10 ng Tat was injected stereotaxically into the CP of rats whose brains were harvested at different time points after the injection (6, 24, 48 h and 1, 2, 4 week, with 4 rats at each time point; total: *n* = 24 rats).

Controls (*n* = 2 for each time point) received saline instead of Tat in the CP (total: *n* = 12 rats). In order to test the specificity of the effects of Tat, 1 μL saline containing 10 ng rat IgG (Sigma) was injected into the CP as a control unrelated protein (*n* = 2 for each time point, total = 12). Saline and saline containing rat IgG were used as negative controls, as was the contralateral side of the unilaterally injected brains.

#### 2.5.2. Injection of SV(Tat)

To assess the effects of more protracted exposure to Tat, we used a system in which Tat is expressed over time in CNS cells. Rats were injected intra-CP with SV(Tat), which allows for continued Tat production by transduced cells. The brains from the animals were then studied 1, 2 and 4 weeks after injection of SV(Tat) for Tat expression and apoptosis. SV(Tat) was injected into the CP of Sprague-Dawley rats (*n* = 5 at each time, total = 15). Controls received an unrelated vector, SV(BUGT), instead of SV(Tat) in the CP (*n* = 5 at each time point, *n* = 15).

#### 2.5.3. Challenge with Tat after Administration of SV(GPx1) or/and SV(SOD1)

To study possible protection by rSV40-mediated overexpression of SOD1 and/or GPx1 from Tat-related injury, we first injected the CP of rats with SV(SOD1) (*n* = 10), SV(GPx1) (*n* = 10) and a mixture 50:50 SV(SOD1)/SV(GPx1) (*n* = 10). One month later, the CP in which SV(SOD1) or/and SV(GPx1) has been administered was injected with 10 ng Tat. Half of the brains were harvested 2 days after injection of Tat into the CP and studied for apoptosis and malondialdehyde (MDA) level at d2 (*n* = 5 for each vector, total = 15 rat). Other brains (*n* = 15) were harvested two weeks after injection of Tat in the CP and studied for neuroinflammation (e.g., immunostaining for microglial cells and astrocytes), In all cases, controls received SV(BUGT) in the CP instead of SV(SOD1), SV(GPx1) or SV(SOD1)/SV(GPx1) (*n* = 10).

### 2.6. In Vivo Injection of Tat and Vectors

Rats were anesthetized with isofluorane UPS (BaxterHealthcare Corp., Deerfield, IL, USA) (1.0 unit isofluorane/1.5 L O_2_ per min) and placed in a stereotaxic apparatus (Stoelting Corp., Wood Dale, IL, USA) for cranial surgery. Body temperature was maintained at 37 °C by using a feedback-controlled heater (Harvard Apparatus, Boston, MA, USA). Glass micropipettes (1.2 mm outer diameter; World Precisions Instruments, Inc., Sarasota, FL, USA) with tip diameters of 15 μm were backfilled with either 5 μL of SV(BUGT), SV(SOD1), SV(GPx1), or a mixture 50:50 SV(SOD1)/SV(GPx1) viral vector, which contains approximately 10^7^ particles. The vector-filled micropipettes were placed in the CP using coordinates obtained from the rat brain atlas of Paxinos and Watson [45]. For injection into the CP, a burr hole was placed +0.48 mm anterior to bregma and −3.0 mm lateral to the sagittal suture. Once centered, the micropipette was placed 6.0 mm ventral from the top of the brain. The same coordinates were used for injecting 10 ng Tat in 1 μL saline, as well as for injecting saline and 1 μL saline containing 10 ng rat IgG. Tat or the vector were given by a Picospritzer II (General Valve Corp., Fairfield, NJ, USA) pulse of compressed N2 duration 10 ms at 20 psi until the fluid was completely ejected from the pipette. Following surgery, animals were housed individually with free access to water and food.

### 2.7. Procedure for Harvesting the Tissue

After a variable survival period, the rats were anesthetized by intraperitoneal injection of sodium pentobarbital (Abbott Laboratories, North Chicago, IL, USA) at 60 mg/kg and perfused transcardially though the ascending aorta with 10 mL heparinized saline followed by ice cold 4% paraformaldehyde (Electron Microscopy Sciences, Fort Washington, PA, USA) in 0.1M phosphate buffer (pH 7.4). Immediately following perfusion-fixation, the rat brains were removed, placed in 4% paraformaldehyde for 24 h, then in a 30% sucrose solution for 24 h, then frozen in methyl butane cooled in liquid nitrogen. Samples were cut on a cryostat (10 μm sections).

### 2.8. Immunocytochemistry

For immunofluorescence, coronal cryostat sections (10 μm thick) were processed for indirect immunofluorescence. Blocking was performed by incubating 60 min with 10% goat, or 10% donkey, serum in phosphate buffer saline (PBS; pH 7.4). Then, sections were incubated with antibodies diluted according to the manufacturer’s recommendations: 1h with primary antibody, then 1 h with secondary antibody diluted 1:100, all at room temperature. Double immunofluorescence was performed as previously described [46]. All incubations were followed by extensive washing with PBS. To stain nuclei, we used mounting medium containing 4′,6-diamidino-2-phenylindole (DAPI) (Vector Laboratories, Burlingame, CA, USA). Specimens were finally examined under a Leica DMRBE microscope (Leica Microsystems, Wetzlar, Germany). Negative controls were performed each time and immunostaining was done, which consisted of preincubation with PBS, a substitution of non-immune isotype-matched control antibodies for the primary antibody, and/or omission of the primary antibody.

### 2.9. Staining of Neurons Using NeuroTrace

Neurotrace (NT) staining has been used as a neuronal marker in studies focusing on the characterization of neurons [47] and NT staining has been performed as previously reported [48,49,50]. After rehydration in 0.1 M PBS, pH 7.4, sections were treated with PBS plus 0.1% Triton X-100 10 min, washed twice for 5 min in PBS then stained by NT (Molecular Probes, Inc., Eugene, OR) (1:100), for 20 min at room temperature. Sections were washed in PBS plus 0.1% Triton X-100 then × 2 with PBS, then let stand for 2 h at room temperature in PBS before being counterstained with DAPI. Combination NT + antibody staining was performed using primary and secondary antibodies staining first (see above), followed by staining with the NT fluorescent Nissl stain. All experiments were repeated 3 times and test and control slides were stained the same day.

### 2.10. TUNEL Assay

Terminal deoxynucleotidyl transferase-mediated biotinylated UTP nick end labeling (TUNEL) assay was performed according to the manufacturer’s recommendations (Roche, Indianapolis, IN, USA) and following a previously described protocol [25]. We used two assays: end-labeling of DNA with fluorescein-dUTP and tetramethylrhodamine-dUTP (TMR-dUTP). To quantitate the TUNEL assay, TUNEL-positive cells were expressed as a total number per CP measured in at least 5 consecutive sections, using the same computerized system as described in the Morphometry section. The final number was an average of results measured in the different sections.

### 2.11. Morphometry

NeuN-, NT-, CD68/ED1-, GFAP-, and Iba-1-positive cells were enumerated manually on the injected and uninjected sides in the whole CP of animals injected with Tat, or saline, in at least 5 consecutive sections using a computerized imaging system (Image-Pro Plus, MediaCybernetics, Bethesda, MD, USA) as previously described [49,51]. In all cases, the final number was an average of results measured in the different sections. This procedure already described for assessment of numbers of transgene-positive cells in the brain [49,52] allows quantitative and relative comparisons among different time points, although it does not reflect the total number of transduced cells *in vivo*. The results were expressed as percentages of NT-positive cells.

Tat- and TUNEL-positive cells were enumerated as previously described in SV(Tat) recipients. Neuronal loss was estimated by calculating the ratio of the number of NT-positive cells on the injected side compared with the number of NT-positive cells on the uninjected side.

### 2.12. Measurement of Malondialdehyde

The measurement of malondialdehyde (MDA) was used as an indicator of lipid peroxidation. The MDA assay was performed using lipid peroxidation kit (Oxford Biochemical Research, Oxford, MI, USA). The assay is based on reaction of chromogenic reagent *N*-methyl-2-phenylindole, with MDA and 4-hydroxyalkenals at 45 °C to form a stable chromophore at 586 nm. MDA standards were prepared with target concentration in reaction mixture ranging from 0 to 4 μM, and the assay was performed following manufacturer’s instructions and as previously reported [53]. Cryostat sections (50 μm thick) of the striatum areas injected with Tat or saline were harvested then homogenized in buffer, and lysates were prepared as previously reported [25,53]. Briefly, for 200 μL of sample, 650 μL of reagent R1 and 150 μL of 12 N HCL were added and incubated at 45 °C for 60 min. The absorbance was measured at 586 nm, and the MDA levels of unknown samples were deduced from the standard curve. MDA levels of samples from CPs injected with Tat after prior administration of SV(BUGT), SV(SOD1), SV(GPx1) or SV(SOD1) and SV(GPx1) were measured the same way.

### 2.13. Statistical Analysis

Comparison of medians between 2 groups was achieved by using the Mann-Whitney test (with a two-tail *p* value). Comparison of medians between more than 2 groups was done by using the Kruskall-Wallis test. The difference between the groups was considered significant when *p* < 0.05. On graphs, values are represented as means ± SEM.

## 3. Results

### 3.1. Injection of Tat Induces Oxidative Damage

**Figure 1:**
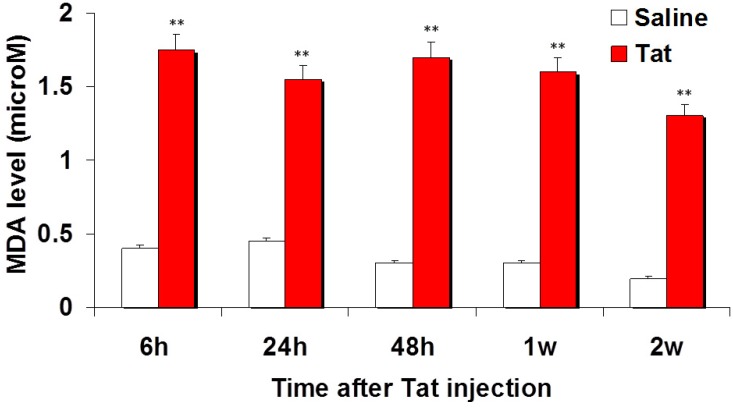
Graph showing the evolution of malondialdehyde (MDA) levels with time after injection of Tat in the caudate-putamen. Levels of MDA increased 6 h after injection of Tat in the CP and remained significantly elevated at the different times considered, even 2 weeks later (** *p* < 0.01).

Because oxidative injury is seen in the brains of patients with HAND, we tested for oxidative damage occurring after Tat injection. Lipid peroxidation was measured by calorimetric MDA assay. Injection of Tat elicited more MDA than did the control saline at the different times considered (*p* < 0.001) (Figure 1).

### 3.2. Injection of Tat into the CP Elicits Apoptosis

**Figure 2:**
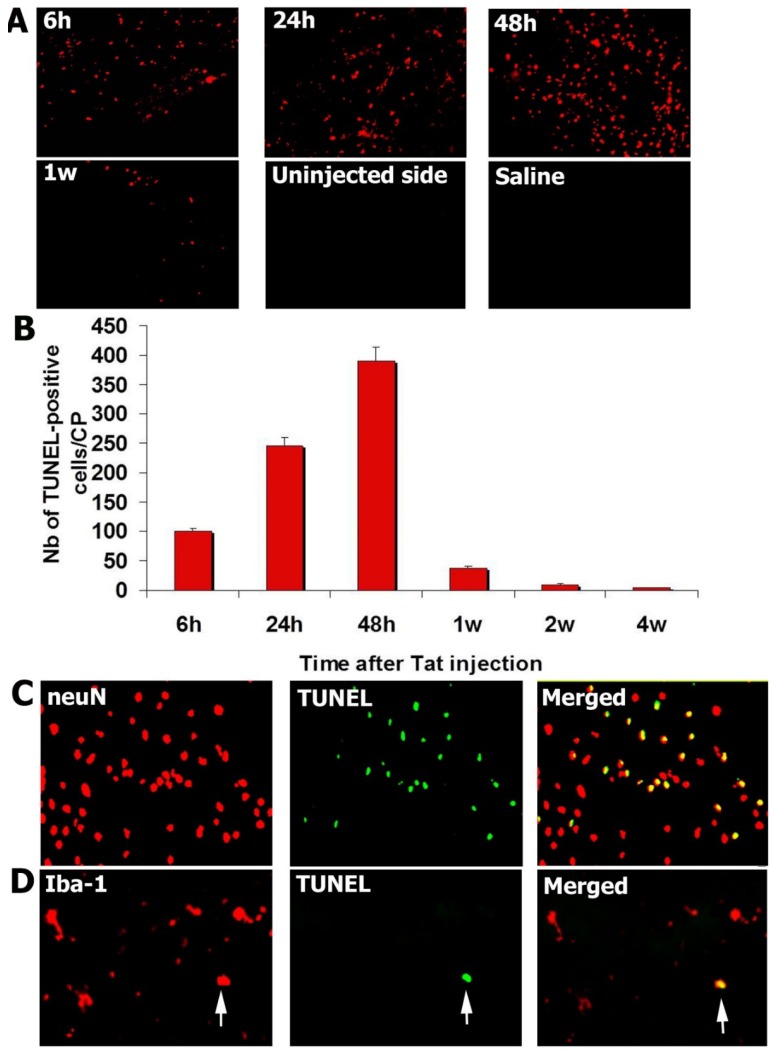
Tat injection in the caudate-putamen induces neuronal apoptosis. (**A**) TUNEL-positive cells were observed in the caudate-putamen (CP) after injection of Tat in the same structure. Number of TUNEL-positive cells peaked 2 days after Tat injection. No TUNEL-positive cells could be seen in CP injected with saline or in the uninjected side; (**B**) Graph showing that TUNEL-positive cells peaked 2 days after injection of Tat in CP; (**C**) TUNEL-positive cells were positive for neuN, a neuronal marker, suggesting that most of apoptotic cells were neurons; (**D**) Rare Iba-1-positive cells were TUNEL-positive (arrow).

After injection of 10 ng Tat into the CP, TUNEL assays were performed to detect apoptosis between 6 h and 4 weeks. TUNEL-positive cells were enumerated in the whole CP, at least in five different sections for each animal. Extremely rare TUNEL-positive cells were seen when the CP was injected with saline or rat IgG (negative controls). Numbers of TUNEL-positive cells peaked 2 days after injection of Tat (Figure 2A,B).

### 3.3. Tat-Induced Apoptotic Cells Are Mainly Neurons

Sections from the CP obtained 2 days after Tat injection were double-stained for TUNEL and neuN, a marker of neurons, or Iba-1, a marker of microglial cells. Most of TUNEL-positive cells were immunopositive for neuN, identifying them as neurons (Figure 2C). Rare TUNEL-positive cells also co-immunostained for Iba-1, a microglial marker (Figure 2D). About 96% of TUNEL-positive cells were neuN-positive, 4% were microglial cells.

### 3.4. HIV-1 Tat Triggers Increases in Microglial Cells and Astrocytes

**Figure 3:**
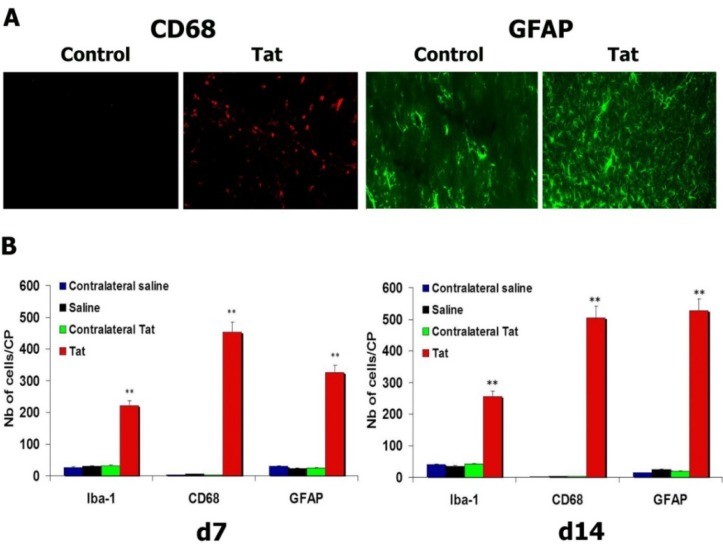
Injection of Tat in the caudate-putamen (CP) triggers neuroinflammation. (**A**) Activated (CD68-positive) microglial cells and proliferating astrocytes were observed in the CP of rats injected with Tat; (**B**) Graph showing an increase in the number of microglial cells (Iba-1 and activated CD68-positive cells) and astrocytes (GFAP-positive cells) persisting til d14. ** *p* < 0.01.

We first characterized different populations of microglial cells by immunocytochemistry to assess if Tat induced an increase of microglial cells. Immunostaining for Iba-1, a marker of quiescent and activated microglial cells, and for CD68/ED1, a marker of activated microglial cells (mainly in the phagocytic state), showed an increase in the numbers of Iba-1- and CD68/ED1-positive cells 7 and 14 days after injection of Tat into the CP. No increase in the number of microglial cells was seen in the contralateral uninjected side or after injection of saline into the CP (Figure 3A,B) (*p* < 0.01: Tat *vs.* saline, both for Iba-1 and CD68/ED1, at d7 and d14). We then enumerated the number of astrocytes by immunostaining for GFAP. An increase in the number of GFAP-positive cells was observed in the CPs injected with Tat. There were more astrocytes 14 days after injection of Tat into the CP than at 7 days (*p* < 0.05). There were significantly fewer astrocytes in the contralateral CP or after injection of saline into the CP (*p* < 0.01: Tat *vs.* saline, at d7 and d14) (Figure 3A,B).

### 3.5. Transgene Expression of Tat by Intra-CP Injection of SV(Tat) Induces Oxidative Stress, Apoptosis and Neuroinflammation

HIV-1 infection of the brain is a long-term disease that is characterized by production of HIV-1 proteins for extended periods of time. We previously reported the use of an SV40-derived expression vector, SV(gp120), to provide ongoing expression of HIV-1 envelope glycoprotein gp120 as a model to study more chronic CNS consequences of protracted production of neurotoxic HIV-1 gene products [53]. We tested whether a similar system could be used to study the effects of Tat production in the CNS for a protracted period of time. We injected SV(Tat) into the CNS. Expression of Tat protein, which is produced as a result of transduction, was demonstrated by immunocytochemistry at different time points after injection of SV(Tat). One month after SV(Tat)injection, Tat-positive cells were still present in the CP. Tat was localized mainly within neurons. The expression of Tat protein was sustained after injection of SV(Tat). However, there was a decrease in the number of Tat-positive cells with time, probably because of the neuronal death following Tat expression. No Tat was detected in the contralateral uninjected side (not shown) or after injection of SV(BUGT), a control vector, into the CP (Table 1).

Apoptotic cells were detected at different times by TUNEL assay in SV(Tat) recipients. Rare TUNEL-positive cells were seen after injection of SV(BUGT) (*p* < 0.001). No apoptotic cells were detected on the contralateral uninjected side (not shown) (Table 1).

The neuronal loss, expressed as a percentage of NT-positive cells compared with the contralateral side, was between 26.7% and 34.3% after injection of SV(Tat) in the CP (7 and 28 days after Tat injection, respectively) and was significantly higher than after injection of SV(BUGT) in the same area (*p* < 0.001) (Table 1).

Lipid peroxidation, measured by calorimetric MDA assay, showed that injection of SV(Tat) induced significantly more MDA than was seen in the recipients of saline control (saline: 0.4 μM *vs.* SV(Tat): 1.4 μM; *p* < 0.01).

**Figure 4:**
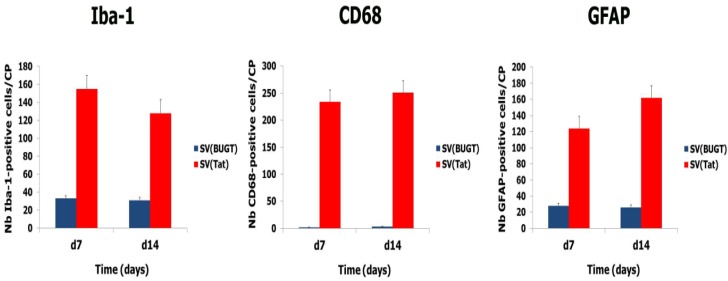
Injection of SV(Tat) in the caudate-putamen (CP) induces neuroinflammation. Brains were harvested one and two weeks after injection of SV(Tat) in the CP. Increased numbers of microglial cells (Iba-1- and CD68-positive cells) and astrocytes (GFAP-positive cells) were observed in the CPs injected with SV(Tat). No inflammation was seen in the CPs injected with the control vector, SV(BUGT).

We enumerated Iba-1-, CD68-, and GFAP-positive cells in SV(Tat) recipients and controls (animals injected with SV(BUGT). Significant higher numbers of microglial cells and astrocytes were observed in the rats administered with SV(Tat), compared to controls (Figure 4), suggesting that continuing production of Tat is associated with neuroinflammation.

**Table 1:** Table showing the changes in the caudate-putamen (CP) of rat following injection of SV(Tat). More numerous Tat-positive cells and TUNEL-positive cells were seen one month after injection of SV(Tat) in the CP (*p* < 0.01: SV(Tat) *vs.* SV(BUGT)). Neuronal loss was observed (*p* < 0.01: SV(Tat) *vs.* SV(BUGT)). An increase in the level of MDA, suggesting of oxidative stress, was also detected (*p* < 0.01: SV(Tat) *vs.* SV(BUGT)). CPs injected with SV(BUGT), an unrelated vector, did not show any modifications.

Days/Tat-Induced Abnormalities	Nb Tat-Positive Cells/Area			Nb TUNEL-Positive Cells/Area			Neuron Loss (% Compared to Contralateral Area)			MDA (μM)		
	SV(BUGT)	SV(Tat)	*P*	SV(BUGT)	SV(Tat)	*P*	SV(BUGT)	SV(Tat)	*P*	SV(BUGT)	SV(Tat)	*P*
d7	-	39.4 ± 4.2	<0.01	2.1 ± 1.8	42.7 ± 4.8	<0.01	1.9 ± 0.1	26.7 ± 3.2	<0.01	NA	NA	-
d14	-	48.7 ± 3.8	<0.01	-	47.2 ± 4.5	<0.01	-	30.8 ± 2.9	<0.01	0.4 ± 0.03	1.4 ± 0.1	<0.01
d28	-	28.5 ± 3.4	<0.01	-	24.6 ± 3.1	<0.01	-	34.3 ± 3.6	<0.01	NA	NA	-

### 3.6. Overexpression of Antioxidant Enzymes Protects against Tat-Induced Lesions

**Figure 5:**
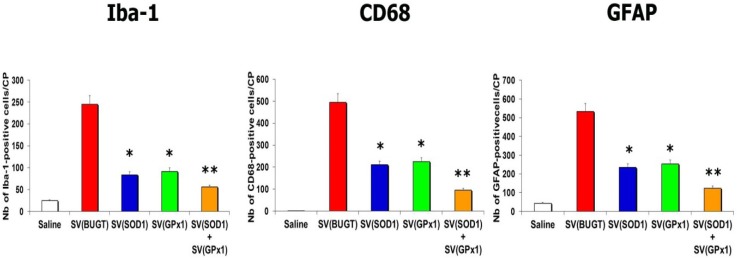
Gene delivery of antioxidant enzymes mitigates Tat-induced neuroinflammation. SV(SOD1), SV(GPx1), or a mixture SV(SOD1)/SV(GPx1), were injected in the caudate-putamen (CP) one month before injection of Tat in the same structure. In the group administered with a control vector, SV(BUGT), increased numbers of microglial cells and astrocytes were seen 2 weeks after injection of Tat. CPs previously injected with SV(SOD1), SV(GPx1), or a mixture SV(SOD1)/SV(GPx1) exhibited significantly fewer microglial cells and astrocytes compared to the group injected with SV(BUGT) then Tat (* *p* < 0.05; ** *p* < 0.01). There were also less-apoptotic cells and no increase in MDA levels in the group injected with saline compared to the group injected with SV(BUGT) then Tat (*p* < 0.01).

To determine whether rSV40-delivered antioxidant enzymes Cu/Zn SOD1 and GPx1 could protect against Tat-induced CNS injury and its consequences, we administered SV(SOD1), SV(GPx1), and a mixture SV(SOD1)/SV(GPx1) to the CP 1 month before we injected Tat. We then assayed for apoptotic cells and oxidative stress 2 days after Tat inoculation. Prior SV40 delivery of antioxidant enzymes reduced Tat-induced lipid peroxidation (Table 2). The reduction in lipid peroxidation was seen in all antioxidant recipient groups, with animals receiving SV(GPx1), SV(SOD1), whether alone or together, this combination being the best protocol. Prior administration of SV(SOD1) and SV(GPx1) also protected the brains from the damage elicited by Tat. That is, there were fewer Tat-induced apoptotic cells in recipients of SV(SOD1) or SV(GPx1). However, the greatest protection from Tat-related neuronal apoptosis was seen when both vectors were administered into the CP together (Table 2). Finally, we harvested brains 2 weeks after Tat injection to assess neuroinflammation. Prior delivery of antioxidant enzymes mitigated Tat-induced neuroinflammation: numbers of Iba-1-, CD68-, and GFAP-positive cells were significantly reduced compared to controls [injection of SV(BUGT)] after injection of SV(SOD1) and/or (SV(GPx1) (Figure 5).

Thus, the injection of SV(Tat) induces substantial expression of Tat in neurons, lipid peroxidation, neuron death, and neuroinflammation.

## 4. Discussion

Advances in the treatment of HIV-1 have dramatically improved survival rates over the past 10 years, but HIV-associated neurocognitive disorder (HAND) remains highly prevalent and continues to represent a significant public health problem, partly because HIV-1 is largely impervious to HAART in the CNS. In the early 1990s, the neurologic complications of HIV-1 infection were classified into two levels of disturbance: (1) HIV-associated dementia (HAD) with motor, behavioral/psychosocial, or combined features; and (2) minor cognitive motor disorder (MCMD). HAD was considered as the most common cause of dementia in adults under 40 [54] and was estimated to affect as many as 30% of patients with advanced AIDS [54], but has become less common since HAART was introduced [55]. This reduction probably reflects better control of HIV in the periphery, since antiretroviral drugs penetrate the CNS poorly. Before the introduction of HAART, most neuroAIDS patients showed subcortical dementia, with predominant basal ganglia involvement, manifesting as psychomotor slowing, Parkinsonism, behavioral abnormalities and cognitive difficulties [56]. MCMD described a less severe presentation of HIV-associated neurocognitive impairment that did not meet criteria for HAD.

More recently, in light of the changing epidemiology of HIV infection, the need to update and further structure the diagnostic criteria for HAND has been recognized [57]. There are several reasons for this update. First, the applicability of the old criteria appears limited in the present age of HAART. Prior to the advent of HAART, a diagnosis of HAD was associated strongly with high viral loads, low T-cell counts, and opportunistic infections. With HAART limiting viral severity, patients with HIV typically live longer with milder medical symptoms. Secondly, guidelines regarding possible neurocognitive impairment due to comorbid conditions with CNS effects (e.g., substance use disorders) were not precisely described in the previous diagnostic scheme. This limitation is particularly important in the era of HAART as those infected with HIV-1 live longer with a host of CNS risk factors, including substance use disorders (e.g., methamphetamine dependence), medical conditions associated with HAART treatment (e.g., hyperlipidemia) and comorbid infectious diseases (e.g., hepatitis C virus) [58].

**Table 2:** Table showing the effect of gene delivery of antioxidant enzymes SOD1 and GPx1 on oxidative stress and apoptosis induced by Tat. Less apoptotic cells were observed in the caudate-putamen (CP) of rats administered with SV(SOD1) and/or SV(GPx1) in the CP before injection of Tat in the same location (*p* < 0.05: SV(SOD1) and SV(GPx1) *vs.* SV(BUGT); *p* < 0.01: SV(SOD1) + SV(GPx1) *vs.* SV(BUGT)). Gene delivery of antioxidant enzymes SOD1 and GPx1 mitigated Tat-induced oxidative stress assessed by MDA levels (*p* < 0.05: SV(SOD1) *vs.* SV(BUGT); *p* < 0.01: SV(GPx1) and SV((SOD1) + SV(GPx1) *vs.* SV(BUGT)).

Vector	Saline	SV(BUGT)	SV(SOD1)	SV(GPx1)	SV(SOD1) + SV(GPx1)
MDA level (μM)	0.38 ± 0.02	1.66 ± 1.5	0.83 ± 0.1	0.42 ± 0.05	0.38 ± 0.04
*P* value	0.01	-	0.05	0.01	0.01
Nb of TUNEL-positive cells/CP	2 ± 0.01	382 ± 41	232 ± 28	255 ± 28	52 ± 6
*P* value	0.01	-	0.05	0.05	0.01

Thus, the newly redefined criteria allow for three possible research diagnoses: (1) asymptomatic neurocognitive impairment (ANI); (2) HIV-associated mild neurocognitive disorder (MND); and (3) HAD. In this classification, the diagnosis of HAND must be determined by assessing at least five areas of neurocognitive functioning known to be affected by HIV infection (e.g., attention/working memory, executive functions, speed of information processing, episodic memory, motor skills, language, and sensoriperception) [57]. Considered as a mild neurocognitive impairment in the absence of declines in everyday functioning, ANI is now estimated to represent the majority of cases of HAND (*i.e.*, about 50% of diagnosed cases) and 21%–30% of the asymptomatic HIV-infected individuals. Detecting patients with mild, yet demonstrable, neurocognitive impairment may help in the effort to pre-identify those at risk for more significant cognitive as well as functional decline, before cognitive deficits contribute to a decline in everyday functioning with serious medical consequences [58]. As effective treatments for neurologic complications are developed, intervention at this earliest stage of HAND might offer the best opportunity to obtain remission, or at least, prevent progression. Formerly referred to as MCMD, mild neurocognitive disorder (MND) requires mild-to-moderate neurocognitive impairment in at least two cognitive domains in addition to mild everyday functioning impairment [57]. Approximately 30%–50% of persons with a HAND diagnosis experience some degree of functional impairment and it is estimated that 20%–40% of HAND diagnoses are of MND, which comprises 5%–20% of the HIV population overall [58]. The most severe form of HAND, HIV-associated dementia (HAD) is marked by at least moderate-to-severe cognitive impairment in at least two cognitive domains along with marked ADL declines that are not fully attributable to comorbidities or delirium [57,58]. Furthermore, HAD represents the most severe form of HAND in terms of its functional impact. After the advent of HAART in the late 1990s, estimates appeared to shift downward with approximately 4% to 7% of persons with AIDS, with more recent appraisals suggesting that as few as 1% to 2% of HIV+ patients meet criteria for HAD [58].

As survival with chronic HIV-1 infection improves, the number of people harboring the virus in their CNS increases, leading to new HIV-1-related neurological manifestations. The prevalence of HAND therefore continues to rise, and less fulminant forms of HAND have become more common than their more severe predecessors [57,58]. HAND remains a significant independent risk factor for AIDS mortality [17,18,19,20,55,57,58]. Incident cases of HAND are accelerating fastest among drug users, ethnic minorities, and women [17,18,19,20]. The number of HIV-infected individuals over 50 years of age is rapidly growing, including patients taking HAART [20]. It has been suggested that in 10 years, 50% of AIDS patients in the United States will be over the age of 50. Moreover, it is becoming clear that the brain is an important reservoir for the virus, and neurodegenerative and neuroinflammatory changes may continue despite HAART [19].

Oxidative stress plays a role in the development of HAND [59,60]. Oxidative stress in HIV-1 dementia has been documented by analyses of brain tissue, including increased levels of lipid peroxidation product (*i.e.*, by immunodetection of 4-hydroxynonenal (HNE) or measurement of malondialdehyde (MDA) levels) and the presence of oxidized proteins (*i.e.*, dinitrophenol (DNP)). Serum levels of GSH and GPx1 are decreased in HIV-1 patients while MDA levels are increased [61]. A characteristic of patients infected with HIV-1 in late stage disease is diffuse intracellular oxidation in the form of decreased availability of GSH, the main cellular antioxidant and redox buffer, and augmented lipid oxidation, which triggers a cascade of downstream signaling events.

Membrane-associated oxidative stress correlates with HIV-1 dementia pathogenesis and cognitive impairment [20]. HNE-positive neurons have been demonstrated in the brains of patients with HIV-1 encephalitis [60,62,63]. In the case of HIV-1 infection, Tat and gp120 can elicit such oxidative stress [20,38,64] which can induce apoptosis in cultured neurons [65]. It can also damage neurons and cause cognitive dysfunction *in vivo* [66]. Tat and gp120 induce ceramide production in cultured neurons by triggering sphingomyelinase activity via a mechanism that involves induction of oxidative stress by CXCR4 activation [20,67]. Oxidative stress can play a role in HAND in other ways as well. Circulating toxins in the CSF, derived from HIV-1-infected cells, may damage mitochondria, leading to release of cytochrome c and then to a cascade of events leading to apoptosis [59,60]. HIV-1 gp120 and Tat can cause free radical production, possibly as part of the signal-transduction pathways they activate [20,64].

It is still unclear whether oxidative stress is the primary initiating event associated with neurodegeneration. However, a growing body of evidence implicates it as being involved in at least the propagation of cellular injury that leads to neuron death [68]. Earlier reports support the hypothesis that oxidative modifications of macromolecular cell components (lipids, proteins and nucleic acids) may be an early step in the mechanism of Tat and gp120 neurotoxicity [36,37].

Microglial cells can proliferate in response to brain injury [69]. Following such insult, reactive microglial cells are mainly proliferating resident cells rather than infiltrating circulating cells [70]. Both populations are different. Infiltrating cells will undergo apoptosis while proliferating resident ones continue to expand [70]. Proliferating resident microglial cells can migrate towards the area of injury too (as infiltrating circulating cells could, but these ones are less numerous and are progressively disappearing because of apoptosis). If neuron loss [24,71,72,73] and astrogliosis [24] have been described in animals receiving gp120 directly into their brains, a temporal relationship between neuronal degeneration, astrocytic reaction and microglial proliferation remained to be established. Thus, we challenged rat CPs with 100–500 ng HIV-1BaL gp120, with or without prior rSV40-delivered superoxide dismutase or glutathione peroxidase [74]. CD11b-positive microglia were increased day 1 post-challenge, while Iba-1- and CD68-positive cells peaked at day 7 and day 14. Moreover, CD68-positive cells exhibited a shape that was close to reactive amoeboid microglial cells. Astrocyte infiltration was maximal at day 7–14. These sequential events were related to the expression of different chemokines and cytokines overtime. It is possible that different populations of microglial cells are recruited following gp120 injection, or that resident microglial cells can be stimulated by the different chemokines/cytokines expressed post-injury (in response to oxidative stress for example), leading to their proliferation and their migration towards the site of injury. SV(SOD1) or SV(GPx1) significantly limited neuroinflammation following gp120 administration into the CP. Thus, gp120 induces neuroinflammation when injected in the rat CP and gp120-induced neuroinflammation correlates with neuron loss [74]. An increase in ROS may play a role in this phenomenon, as evidenced by the protective effects of rSV40-delivered antioxidant enzymes. The results we observed here by injecting Tat in the CP are somehow comparable to those seen with gp120.

Free radical production may be accompanied by elevated expression of MIP-1 alpha contributing to microglial recruitment and delayed neuronal death in several models of CNS injury [75,76]. The radical scavengers, like vitamin E analogs, may inhibit free radicals and MIP-1 alpha production, and recruitment of microglia in the injured area [77]. In models of ischemia/reperfusion injury, transgenic mice that overexpressed antioxidant enzymes, such as SOD-1 and GPx1 showed less upregulation of MIP-1 alpha and MCP-1 and less neuron loss and inflammation [78]. Concerning HIV-1-related neurotoxicity, it has been recently shown that both gp120 and methamphetamine (MA) induce ROS production in concentration- and time dependant manners. Moreover, the involvement of cytochrome P 450 (CYP) and NADPH-oxidase (NOX) pathways in gp120/MA-induced oxidative stress and apoptotic cell death in astrocytes has been reported [79]. Tat can up-regulate cytokines (*i.e.*, IL-6, IL-8) expression in breast cancer cells [80], and can also increase the expression of the chemokine CCL5 in astrocytes [81]. Additionally, Tat-induced production of chemokine (MCP-1) and cytokines (TNF-α, IL-6) in astrocytes is increased by morphine, through NF-κB trafficking and transcription [82]. Our findings extend the principle of antioxidant protection from neuroinflammation to HIV-related injury, and suggest that rSV40 antioxidant gene delivery may be therapeutically applicable in the case of ongoing injury and neuroinflammation such as HAND.

A few antioxidants have been tried in small prospective controlled studies in HAND. However, the findings have all been relatively disappointing so far. Selegiline (l-deprenyl), which mechanism of action is speculative, albeit it might decrease the production of ROS and serve as an anti-apoptotic factor, was used in 2 double-blind controlled studies in the pre-HAART era. The first trial involving patients with minor cognitive and motor dysfunction (MCMD) showed improvement in verbal learning and trends for improvement in recall [83,84]. The second study was a smaller study in patients with MCMD and HIV dementia and showed significant improvement in delayed recall. However, other tests were not improved. A slight improvement was noted in patients treated with OPC-14117, a lipophilic compound structurally similar to vitamin E that acts as an antioxidant by scavenging superoxide radicals [85]. CPI-1189, a lipophilic antioxidant that scavenges superoxide anion radicals and block the neurotoxicity of gp120 and TNF-α [86], showed no effect on neurocognition in patients with MCMD and HIV dementia [87].

Upstream and downstream antioxidant therapeutic approaches in HAND have previously been discussed [88]. Upstream preventive treatment is based on prevention of free radical generation, regulation of neuronal protein interaction with redox metals (*i.e.*, Fe) and maintaining normal cellular metabolism. Our daily diet contains several natural antioxidants (lipoic acid, and vitamins E). Antioxidant therapy involving endogenous enzymes and some anti-inflammatory drugs constitute upstream therapy in ROS generation and can prevent downstream neurodegeneration. Vitamin E can block the neurotoxicity induced by CSF of patients with HIV dementia [60]. Flavinoids are a group of compounds made by plants that have antioxidant and neuroprotective properties. This class of molecules has weak estrogen-receptor-binding properties and thus, do not have the side effects of estradiol. It has been described that diosgenin, a plant-derived estrogen present in yam and fenugreek can prevent neurotoxicity by HIV-1 proteins and by CSF from patients with HIV dementia [60]. *N*-acetyl-l-cysteine (NAC) is a nutritional supplement precursor in the formation of the antioxidant glutathione in the body and its sulfhydryl group confers antioxidant effects and is able to reduce free radicals. NAC injected intraperitoneally into rodents increases glutathione levels in the brain and protects the CNS against the damaging effects of hydroxyl radicals and lipid peroxidation product acrolein [89]. However, NAC itself does not cross the BBB easily. *N*-acetylcysteine amide (NACA), a modified form of NAC, where the carboxyl group has been replaced by an amide group, has been found to be more effective in neurotoxic cases because of its ability to permeate cell membranes and the blood-brain barrier (BBB). Treatment of animals injected intravenously with gp120, Tat and methamphetamine METH by NACA significantly rescued the animals from oxidative stress. Further, NACA-treated animals had significantly less BBB permeability as compared to the group treated with gp120 + Tat + METH alone, indicating that NACA can protect the BBB from oxidative stress-induced damage in gp120, Tat and METH exposed animals [90].

The therapeutic coverage of post oxidative stress events can be done by downstream antioxidant therapy. Non steroidal anti-inflammatory drugs (NSAIDS) limit the infiltration of macrophages and can reduce the inflammatory cascade induced by oxidative stress. Minocycline is a tetracycline-derived compound that demonstrated neuroprotective profile in several models of neurodegeneration. The molecule has significant anti-inflammatory actions and can easily cross the BBB. Estradiol can protect against the neurotoxic effects of HIV-1 proteins in human neuronal cultures, probably by protecting the neuronal mitochondria in a receptor-independent manner [60]. However, estradiol has well known side effects in women (potential risk of developing breast or uterine cancer), and cannot be used in men or children because of feminizing effects. It has been shown that several novel antioxidants (ebselen, diosgenin) can protect *in vitro* against neurotoxicity induced by CSF from patients with HIV dementia [60].

It is likely that neuroprotective therapies should benefit from multiple and combination approaches targeting different aspects and pathways of the oxidative-stress insult. For example, coupling a potent antioxidant with a compound that modifies downstream signaling pathways (*i.e.*, minocycline) could provide a synergistic neuroprotective effects, at lower doses (and thus with less toxicity) that each molecule could achieve alone. The combination of HAART with an antioxidant compound and a molecule involved in downstream antioxidant therapy could be a promising avenue in the treatment of HAND. However, it should be reminded that one of the challenges in designing antioxidants to protect the CNS against ROS is the crossing of the BBB.

Experimental systems to study how gp120 and other HIV proteins affect the brain are limited to the acute effects of recombinant proteins *in vitro* or *in vivo*, or to Simian Immunodeficiency Virus (SIV)-infected monkeys. To circumvent these limitations, we have described an experimental rodent model of ongoing gp120-induced neurotoxicity in which HIV-1 Env is expressed in the brain using a SV40-derived gene delivery vector, SV(gp120) [53]. Inoculated stereotaxically into the rat CP, SV(gp120) causes a lesion in which neuron and other cell apoptosis continues for at least 12 weeks. HIV gp120 was expressed throughout this time. SV(gp120)-induced lipid peroxidation was documented by both MDA and HNE assays. Thus, *in vivo* inoculation of SV(gp120) into the rat CP causes ongoing oxidative stress and apoptosis in neurons and so may represent a useful animal model. We also tested the effect of SV(gp120) on neuroinflammation. Increase in microglia and astrocytes was seen following intra-CP SV(gp120) injection, suggesting that continuing gp120 production increased neuroinflammation, as well as microglia and astrocytes proliferation after injection of SV(gp120) in the striatum. However, SV(gp120)-induced inflammation was reduced by prior gene transfer of antioxidant enzymes. Thus, gp120-induced CNS injury, neuron loss and inflammation may be mitigated by antioxidant gene delivery. Similar results using SV(Tat) are shown in the present work.

We sought here to characterize Tat-induced neuroinflammation in different experimental settings and to test an ability to limit the extent of the inflammatory process by rSV40 gene delivery of antioxidant enzymes. The results presented here are somehow comparable to the ones observed with gp120. SV(Tat) induces protracted expression of Tat, mainly in neurons, oxidative stress, neuronal apoptosis, neuroinflammation. Further studies should be undertaken to dissect the role of Tat in oxidative stress; for example, it would be worth measuring free radicals (particularly hydrogen peroxide) after Tat injection.

## 5. Conclusions

HIV-1-associated neurocognitive disorder (HAND) is an increasingly common and progressive disease, characterized by progressively deteriorating CNS function. HIV-1 gene products, particularly gp120 and Tat, elicit ROS that lead to oxidant injury and causes neuron apoptosis, as well as subsequent consequences (e.g., neuroinflammation, abnormalities of the BBB). The understanding of, and developing therapies for HAND requires accessible models of the disease. We have devised experimental approaches to studying the acute and chronic effects of gp120 and Tat on the CNS. Even though HIV-1 gp120 and Tat cannot be solely responsible for the pathogenesis of HAND, the animal models used in the present study are able to recapitulate many aspects of HAND. These approaches to gp120 and Tat administration may therefore represent useful animal models for studying the pathogenesis and treatment of HIV-1 gp120- and Tat-related damage. Gene delivery of antioxidant enzymes by recombinant SV40-derived vectors protects against gp120 and Tat-induced oxidative stress and neuronal apoptosis, opening new avenues for potential therapeutics of HAND.
